# Insight into Glutamatergic Involvement in Rewarding Effects of Mephedrone in Rats: In Vivo and Ex Vivo Study

**DOI:** 10.1007/s12035-021-02404-y

**Published:** 2021-05-21

**Authors:** Olga Wronikowska, Maria Zykubek, Agnieszka Michalak, Anna Pankowska, Paulina Kozioł, Anna Boguszewska-Czubara, Łukasz Kurach, Artur Łazorczyk, Katarzyna Kochalska, Sylwia Talarek, Tymoteusz Słowik, Radosław Pietura, Joanna Kurzepa, Barbara Budzyńska

**Affiliations:** 1 Department of Medical Chemistry, Medical University of Lublin, Chodzki 4a Street, 20-093 Lublin, Poland; 2 Independent Laboratory of Behavioral Studies, Chair and Department of Medical Chemistry, Medical University of Lublin, Chodzki 4a Street, 20-093 Lublin, Poland; 3 Department of Radiography, Medical University of Lublin, Staszica 16 Street, 20-081 Lublin, Poland; 4 Department of Pharmacology and Pharmacodynamics, Medical University of Lublin, Chodzki 4a Street, 20-093 Lublin, Poland; 5 Centre of Experimental Medicine, Medical University of Lublin, Jaczewskiego 8 Street, 20-090 Lublin, Poland; 6 I Department of Medical Radiology, Medical University of Lublin, Jaczewskiego 8 Street, 20-090 Lublin, Poland

**Keywords:** Place preference, Mephedrone, Glutamate, MRS, Chromatography

## Abstract

Mephedrone is a widely used drug of abuse, exerting its effects by interacting with monoamine transporters. Although this mechanism has been widely studied heretofore, little is known about the involvement of glutamatergic transmission in mephedrone effects. In this study, we comprehensively evaluated glutamatergic involvement in rewarding effects of mephedrone using an interdisciplinary approach including (1) behavioural study on effects of memantine (non-selective NMDA antagonist) on expression of mephedrone-induced conditioned place preference (CPP) in rats; (2) evaluation of glutamate concentrations in the hippocampus of rats following 6 days of mephedrone administration, using in vivo magnetic resonance spectroscopy (MRS); and (3) determination of glutamate levels in the hippocampus of rats treated with mephedrone and subjected to MRS, using ion-exchange chromatography. In the presented research, we confirmed priorly reported mephedrone-induced rewarding effects in the CPP paradigm and showed that memantine (5 mg/kg) was able to reverse the expression of this effect. MRS study showed that subchronic mephedrone administration increased glutamate level in the hippocampus when measured in vivo 24 h (5 mg/kg, 10 mg/kg and 20 mg/kg) and 2 weeks (5 mg/kg and 20 mg/kg) after last injection. Ex vivo chromatographic analysis did not show significant changes in hippocampal glutamate concentrations; however, it showed similar results as obtained in the MRS study proving its validity. Taken together, the presented study provides new insight into glutamatergic involvement in rewarding properties of mephedrone.

**Supplementary Information:**

The online version contains supplementary material available at 10.1007/s12035-021-02404-y.

## Introduction

Mephedrone (RS)-1-(4-methylphenyl)-2-metyloaminopropan-1-one (also known as 4-metylometcatynon, 4-MMC, M-CAT) is a synthetic derivative of cathinone, and its main compound is found in *Catha edulis*, a plant naturally grown mainly in East Africa [1]. Mephedrone represents the group of novel psychoactive substances (NPS) which consist of compounds designed to mimic existing established recreational drugs [2]. The online availability of mephedrone along with its low price and worsening quality of other drugs (e.g. 3,4-methylenedioxymethamphetamine (MDMA) and cocaine) led to an increase in mephedrone consumption in Europe and the USA between 2009 and 2010, being a serious health hazard since then [3]. Mephedrone exerts its effects by interacting with plasma membrane monoamine transporter proteins for dopamine (DA) (dopamine transporter, DAT), noradrenaline (NA) (noradrenaline transporter, NET) and serotonin (5-HT) (serotonin transporter, SERT) and by increasing the levels of all above-mentioned monoamines in the central nervous system [4–6]. Mephedrone causes a rapid and dose-dependent increase in both 5-HT and DA levels in the nucleus accumbens (NAc) [7, 8], striatum and frontal cortex in rats [7]. Moreover, it has been shown that repeated mephedrone administration in binge-like regimen causes a rapid decrease in striatal DA and hippocampal 5-HT transporter function in mice which can be perceived as a neurotoxic effect [9]. However, another study showed that mephedrone does not cause damage to striatum long-lasting hippocampal 5-HT and DA nerve endings in mice. Nevertheless, mephedrone enhances the neurotoxic effects of amphetamine and MDMA on DA nerve endings [10, 11]. It has been reported that mephedrone also does not cause toxicity to 5-HT nerve endings of the hippocampus [12]; however, it can cause acute but not lasting 5-HT depletion [5, 13]. The effects of mephedrone can be potentiated because of its metabolites, which display a similar activity as mephedrone and an ability to interact with monoamine transporters, resulting in inhibition of their reuptake [14].

Although the mechanism of action of mephedrone regarding its interaction with monoamine transporters has been widely studied and well described, the knowledge of the involvement of glutamatergic transmission in mephedrone effects is still limited. Glutamate is the major excitatory neurotransmitter in the mammalian brain, acting through many different receptors that can be divided into two main groups: ionotropic glutamate receptors (iGluRs, fast-acting ligand-gated ion channels) and metabotropic glutamate receptors (mGluRs, slow-acting G-protein-coupled receptors) [15, 16]. Group of iGluRs consists of *N*-methyl-d-aspartate (NMDA), α-amino-3-hydroxy-5-methyl-4-isoxazole propionic acid (AMPA) and kainate receptors [17]. Regarding mGluRs, eight subtypes have been identified (mGluR1–mGluR8) so far, which can be divided into three groups depending on their pharmacological selectivity and signal transmission pathways [15].

Since there is strong evidence that glutamate is involved in drug-induced addiction and reward [18, for review], the aim of this study was to evaluate the involvement of glutamatergic transmission in rewarding effects of mephedrone. To achieve this goal, behavioural study, as well as in vivo imaging techniques and ex vivo biochemical analysis, was performed. For the behavioural studies, a well-established paradigm of conditioned place preference (CPP) was used with a subsequent assessment of the effects of memantine, a non-competitive glutamatergic receptors antagonist, on the expression of mephedrone-induced CPP. Since there are limited and incomplete data on glutamate levels in specific brain areas after mephedrone administration [19–21], the next step of the study was to evaluate glutamate levels, both in vivo and ex vivo, in the hippocampus which is, along with the mesocorticolimbic reward system, one of the major structures associated with the development of addiction.

**Fig. 1:**
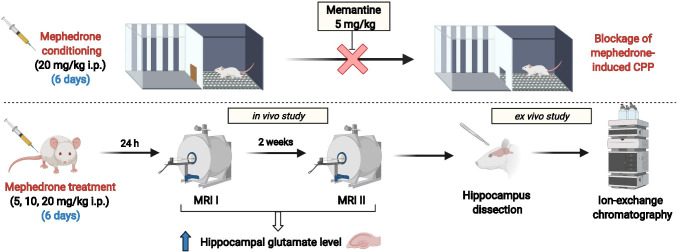
A summary of research conducted in the presented study. This figure was created with BioRender.com

We used two complementary (ex vivo and in vivo) methods. First, the in vivo magnetic resonance spectroscopy (MRS) was performed to measure above-mentioned dose-dependent as well as time-dependent changes in the hippocampal glutamate level after 6 days of mephedrone administration. MRS is a sensitive and advanced technique that provides information on the biochemical composition of brain tissue in a non-invasive way. For a decade, 1H MRS has become an ideal tool for observing changes in the concentration of metabolites in disease entities in the brain, allowing detection of metabolites such as *N*-acetylaspartate, choline, myo-inositol, creatine, glutamate, glutamine, gamma-aminobutyric acid and lactate [22, 23]. However, to prove the validity of this method for further usage in behavioural research, we verified MRS measurements with the ion-exchange chromatographic method and performed ex vivo biochemical assessment of glutamate hippocampal levels in rats subjected to two MRS measurements after 6 days of mephedrone administration. Taken together, presented results provide complex and comprehensive insight into glutamatergic involvement in rewarding properties of mephedrone. The experimental design of research conducted in the presented study is presented in Fig. 1.

## Materials and Methods

### Animals

The experiments were carried out on drug-naive male (8 weeks old, weighing 200–250 g) Wistar rats obtained from the Centre of Experimental Medicine of the Medical University of Lublin. Each experimental group consisted of 8 rats (for the CPP paradigm) or 9 rats (for the MRS and subsequent chromatographic measurements). The animals were kept under standard laboratory conditions (12-h light/dark cycle, lights on 8.00 a.m., room temperature of 21 ± 1 °C, relative humidity of 50 ± 5%) with free access to tap water and a laboratory chow (Agropol, Poland). Animals were housed in pairs with weight-matched conspecific. All experiments were carried out between 8 a.m. and 4 p.m.

### Ethics Statement

All experiments were conducted according to the National Institute of Health Guidelines for the Care and Use of Laboratory Animals and to the European Community Council Directive for the Care and Use of Laboratory Animals of 22 September 2010 (2010/63/EU). The protocol was approved by the local ethics committee (Permit Number: 53/2018). All experiments were conducted having regard to minimising potential pain, suffering or distress of animals.

### Drugs

The following compounds were used: mephedrone hydrochloride (Tocris Bioscience, Cat. No. 4443) and memantine hydrochloride (Tocris Bioscience, Cat. No. 0773/50). For the CPP paradigm, mephedrone (20 mg/kg) was administered for 6 consecutive days of conditioning (once a day, immediately before the afternoon session), and for MRI study (followed by the chromatographic analysis), mephedrone (5 mg/kg, 10 mg/kg and 20 mg/kg) was administered once a day for 6 consecutive days preceding first imagining. To assess the effect of memantine (2.5 mg/kg and 5 mg/kg) on the expression of mephedrone-induced CPP, this drug was administered once (30 min prior to testing). Drugs were administered in intraperitoneal (i.p.) injections at a volume of 2 ml/kg. Control groups received saline solutions (0.9% NaCl) via the same route of administration and at the same volume. The solutions were freshly made each day of the experiment. Chosen drug doses were based on preliminary studies, which showed that mephedrone at the dose of 20 mg/kg induced the most robust response in the CPP paradigm; therefore, this dose has been chosen for the evaluation of glutamatergic involvement in rewarding properties of mephedrone in the presented research. For the MRI study, rats were anaesthetised with a mixture of 3.5% isoflurane and 100% oxygen at the flow level of 0.7 l/min and remained anaesthetised throughout the study on the lowest possible dose of isoflurane (approximately 2%).

### Experimental Procedure and Treatment

#### CPP Paradigm

##### CPP Apparatus and Software

CPP procedure was conducted using Ugo Basile CPP system and VideoMot software. Single Ugo Basile apparatus (external dimensions: 63 cm × 32 cm × 35 cm) consists of two compartments (internal dimensions: 30 cm × 30 cm × 30 cm) differing by tactile and visual stimulation (floor structures and wall patterns) which are divided by the guillotine doors. One compartment has black and white striped walls and floor with round 2-mm holes, and the other one has black walls and floor with square 10 mm × 10 mm holes. The place conditioning experiment was conducted in the unbiased design in which animals do not show any initial preference to either of the compartments. VideoMot software enables live tracking of the animal by 3-point detection (head/centre/tail) by a contrast filter which allows it to measure time spent in each compartment, as well as the distance travelled.

##### CPP Procedure

**Fig. 2:**
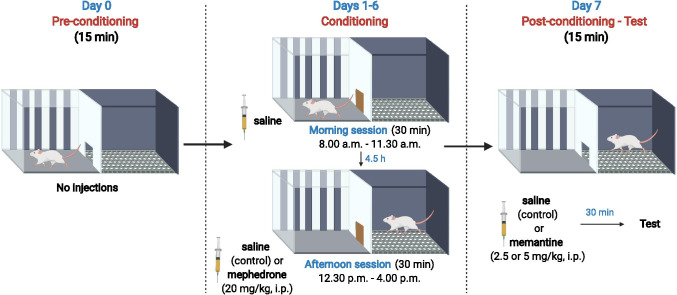
Experimental scheme of CPP procedure in the assessment of glutamatergic involvement in mephedrone-induced CPP. This figure was created with BioRender.com

CPP consisted of 3 phases: pre-conditioning, conditioning and post-conditioning (the test). One day prior to the CPP procedure, animals were habituated, for 15 min, to the apparatus to minimise stress which could affect the behavioural response. The CPP procedure was already validated in our laboratory and conducted as previously described in detail [24–26] with a small modification of using 2-compartment apparatus in comparison to previously used 3-compartment apparatus. In day 0, during pre-conditioning, the guillotine door was open and animals had free access to both compartments. Initial preference was measured. In days 1–6, the guillotine door was closed. Within each group, animals were randomly divided into 2 groups conditioned in different compartments. In the morning session, each animal was confined in one compartment, whereas in the afternoon session, each rat was confined in the other compartment. Animals received the saline injection in the morning session. Immediately before the afternoon session, animals received injections of saline or mephedrone (20 mg/kg). Morning and afternoon sessions were separated by a 4-h interval. In day 7, saline or memantine (2.5 mg/kg or 5 mg/kg) was administered 30 min prior to the testing. The guillotine door was open. Animals had free access to both compartments, and post-conditioning preference was measured. The experimental scheme of CPP procedure is presented in Fig. 2.

#### Locomotor Activity

Regarding the fact that animals’ mobility can influence results observed in CPP, locomotor activity was also evaluated. Since the goal of our study was to assess whether administered drugs affected animals’ locomotion during post-conditioning test, total horizontal activity (distance travelled in metres) was recorded for 15 min on the test day (24 h after last mephedrone administration and 30 min after memantine administration). The measurements of this parameter were performed during test day using the Ugo Basile CPP system and VideoMot software. The possibility of simultaneous measurement of CPP values and locomotor activity values is an undeniable advantage of this protocol as it does not expose animals to additional stress.

#### Magnetic Resonance Study

For MR study, thirty-six rats were divided into 4 study groups, receiving saline or mephedrone at the dose of 5 mg/kg, 10 mg/kg and 20 mg/kg (*n* = 9 in each group) for 6 consecutive days before the first examination. The animals were weighed and food deprived for 6 h before each scan. Two separate MR spectroscopy examinations of the animals were obtained with an interval of 2 weeks between the first and second measurements. To assess the effectiveness of anaesthesia, breathing was monitored ~ 40–50 bpm. Body temperature was maintained about 37 °C using circulating water and was verified using warm water in a closed circuit (Small Animal Instruments, Inc., NY, USA).

**Fig. 3:**
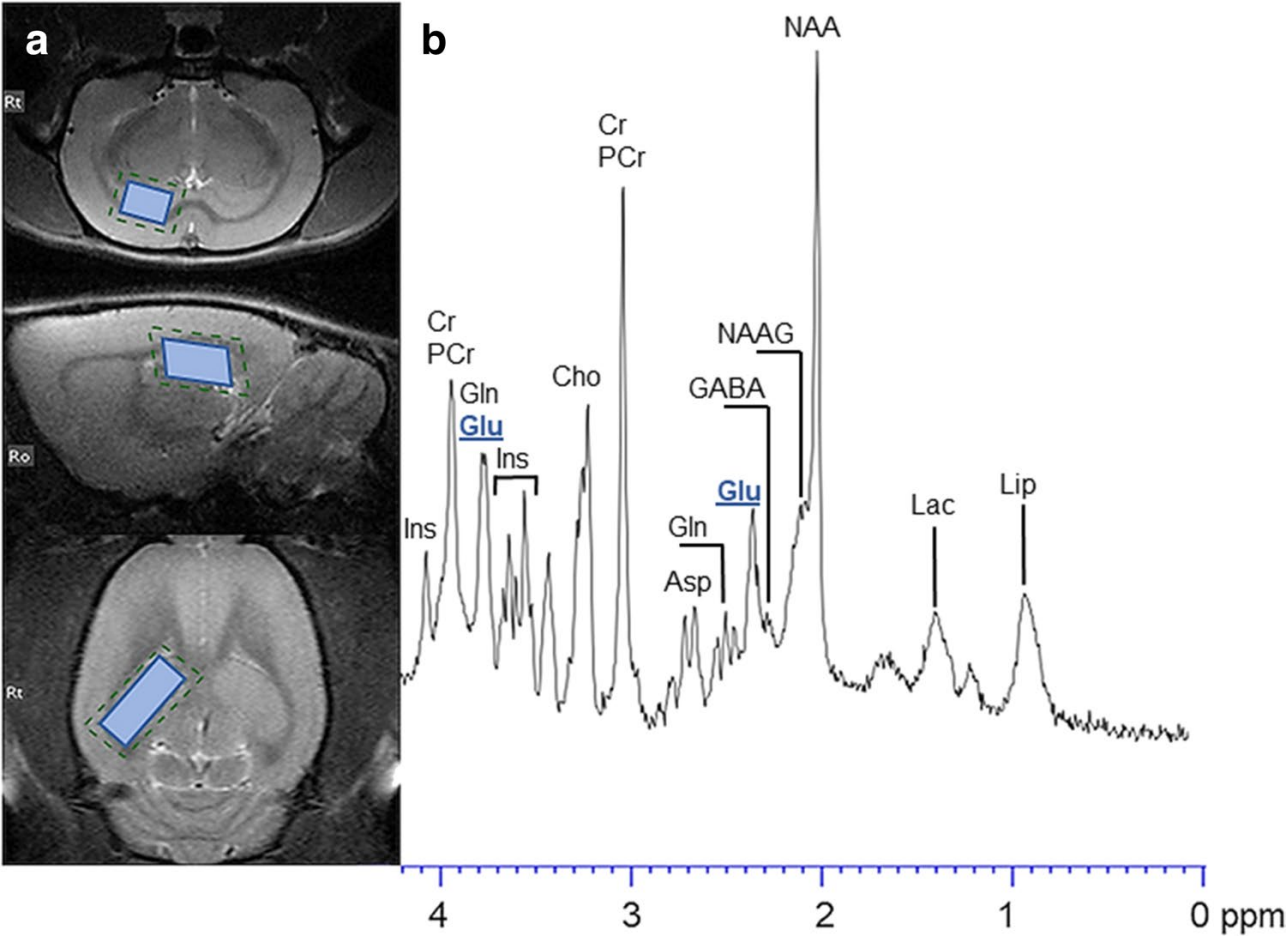
The location of the voxel in the rat’s right hippocampus marked on MR images in sagittal, axial and coronal planes (**a**). A single in vivo MRS spectrum acquired using the PRESS sequence in MR 7 T (**b**). Compounds contained in the hippocampus included in spectrum are *N*-acetylaspartate (NAA), *N*-acetylaspartylglutamate (NAAG), gamma-aminobutyric acid (GABA), glutamate (Glu), glutamine (Gln), aspartate (Asp), creatine (Cr), phosphocreatine (PCr), choline (Cho), myo-inositol (Ins), lactate (Lac) and lipid (Lip)

Magnetic resonance spectroscopy was performed on an MR 7 T horizontal bore magnet (70/16 PharmaScan, ParaVision 6.0.1; Bruker BioSpin GmbH, Germany) using a 72-mm-inner diameter volume coil for transmitting and a 20-mm surface loop coil for receiving. The whole study of one rat lasted about 2.5 h. Morphological images were acquired using T2-weighted rapid acquisition with refocused echo sequence (RARE) (TR/TE = 2500/33 ms, matrix = 256 × 192, slice thickness = 0.8 mm, rare factor = 8, averages = 1). Using high-quality structural brain images, a volume of interest (VOI) was placed in the right hippocampus, with a size of VOI = 2 mm × 2 mm × 5 mm (20 µl). Magnetic field shimming procedure was performed using the built-in Paravision MAPSHIM routine (Bruker BioSpec, Ettlingen, Germany), resulting in full width at half maximum (FWHM) about 8–9 Hz. Proton MRS spectra were measured by the point resolved spectroscopy sequences (PRESS) using TR = 2500 ms, TE = 16 ms, averages = 1024, repetition = 1 and acquisition points = 2048 (Fig. 3). The water signal was suppressed by variable pulse powers and optimised relaxation delays (VAPOR). VAPOR interpulse delays and pulse amplitudes were manually adjusted to each animal to achieve optimal water suppression. MR spectra were analysed using LCModel™ (Linear Combination of Model Spectra) software (version 6.3–1). In this study, LCModel™ was employed in the standard configuration with the analysing window from 0.2 to 4 ppm. The unsuppressed water signal measured from the same VOI was used as an internal reference for the absolute quantification of metabolites.

#### Chromatographic Determination of Glutamate Concentrations

After the second MRI measurement, animals were decapitated and the whole brain was carefully taken out and rinsed in isotonic saline to remove blood. The hippocampus was rapidly dissected and used for the study. To evaluate glutamate concentration, hippocampi were homogenised and deproteinised in 6% sulphosalicylic acid in lithium citrate buffer (pH 2.6) in 1:10 ratio. Then, the samples were centrifuged (20 min at 12,000 rpm) and such obtained supernatants were used for glutamate determination with the use of ion-exchange chromatography on an INGOS AAA 5000 apparatus for automatic analysis of amino acids (Ingos Corp., Czech Republic). Amino acids were separated using analytic column Ostion LG FA (Ingos Corp., Czech Republic) and five lithium citrate buffers (pH 2.6, 3.1, 3.35, 4.05 and 4.65, respectively). Chromatograms were evaluated for glutamate concentration with the use of the original software Clarity 8.1 (DataApex, Czech Republic).

### Statistical Analysis

The data were analysed by the one- or two-way analysis of variance (ANOVA) with multiple comparisons, and the post hoc comparisons of means were carried out with Tukey’s test, when appropriate. For the CPP paradigm, the data was analysed using two-way ANOVA with multiple comparisons. The substance used for conditioning (saline/mephedrone 20 mg/kg) was considered as one of the defining factors in the two-way ANOVA, whereas the dose of memantine (0 mg/kg, 2.5 mg/kg or 10 mg/kg), administered during post-conditioning test, was considered as the second defining factor. For the CPP test, data are expressed as means ± standard deviation (SD) of scores (i.e. the differences between post-conditioning and pre-conditioning time spent in the drug-associated compartment). For the evaluation of locomotor activity, data were analysed using two-way ANOVA with multiple comparisons, with analogous defining factors as described for the CPP paradigm. The data of locomotor activity are expressed as means ± SD of distance travelled measured in metres for 15 min during the post-conditioning test. For the MRS study and the chromatographic determination of glutamate concentration, data were analysed using one-way ANOVA with multiple comparisons and the dose of mephedrone used for the conditioning (0 mg/kg, 5 mg/kg, 10 mg/kg or 20 mg/kg) was chosen as a defining factor. The data of MRS are expressed as means ± SD of glutamate concentrations (mM), and for the chromatographic determination, the data are expressed as means ± SD of glutamate concentrations (µM/g tissue). All statistical tests were performed using GraphPad Prism (version 8.0.1) for Windows (GraphPad Software, USA). The confidence limit of *p* < 0.05 was considered statistically significant.

## Results

### Effects of Memantine on Expression of Mephedrone-Induced CPP

**Fig. 4:**
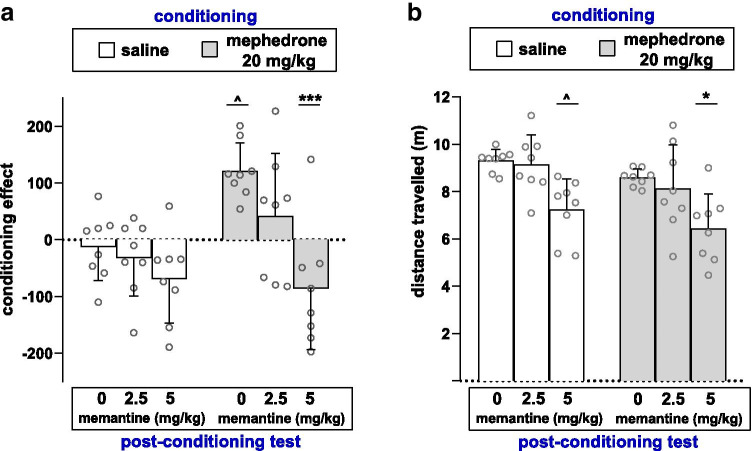
Effects of memantine on mephedrone-induced CPP (**a**) and locomotor activity (**b**). Data represent means ± SD and are expressed as **a** the difference (in s) between post-conditioning and pre-conditioning time spent in the drug-associated compartment and **b** the distance travelled (in m) during the test day; *n* = 8 rats per group; ^*p* < 0.05 vs. saline-conditioned animals treated with saline during the test day; *p < 0.05, ***p < 0.001 vs. mephedrone-conditioned animals treated with saline during the test day (Tukey’s test)

Figure 4a indicates the effect of memantine on the expression of mephedrone-induced CPP in rats (two-way ANOVA: memantine treatment: *F* (2, 42) = 10.57, *p* = 0.0002; mephedrone conditioning: *F* (1, 42) = 7.336, *p* = 0.0097; interaction: memantine treatment × mephedrone conditioning: *F* (2, 42) = 3.480, *p* = 0.0399). Firstly, post hoc Tukey’s test confirmed priorly reported mephedrone-induced rewarding effects, showing that mephedrone (20 mg/kg) induced CPP when compared to saline-conditioned rats (*p* < 0.05). Furthermore, post hoc analysis indicated that administration of memantine (5 mg/kg) during the test day significantly decreased score value in mephedrone-conditioned rats as compared to mephedrone-conditioned rats, treated with saline on the test day (*p* < 0.001).

### Evaluation of Locomotor Activity

Figure 4b indicates the effects of mephedrone and memantine treatment on locomotor activity in rats (two-way ANOVA: memantine treatment: *F* (2, 42) = 13.79, *p* < 0.0001; mephedrone conditioning: *F* (1, 42) = 5.670, *p* = 0.0219; interaction: memantine treatment × mephedrone conditioning: *F* (2, 42) = 0.05344, *p* = 0.9480). Post hoc Tukey’s test showed that the administration of memantine (5 mg/kg) during the test day decreased the distance travelled in saline- and mephedrone-conditioned rats as compared to saline- and mephedrone-conditioned groups, respectively, treated with saline on the test day (*p* < 0.05).

### MRI Results of Glutamate Concentrations in the Hippocampus in Mephedrone-Treated Rats

**Fig. 5:**
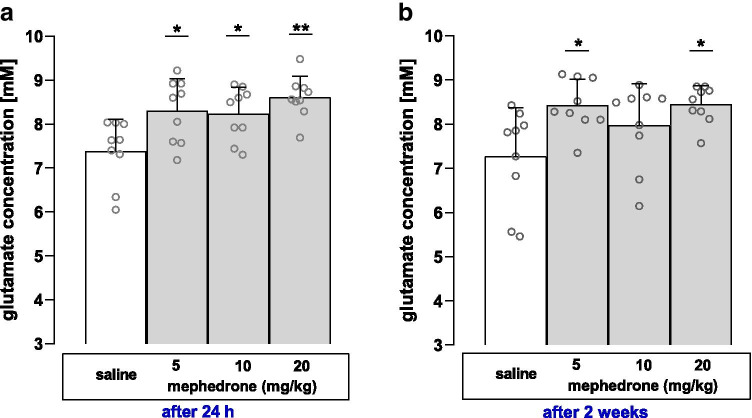
Glutamate concentration levels in the hippocampus after 6 days of mephedrone (5–20 mg/kg) administration, evaluated with MRS and measured 24 h (**a**) or 2 weeks (**b**) after last mephedrone administration. Data represent means ± SD and are expressed as glutamate concentration (mM); *n* = 9 rats per group. *p < 0.05, **p < 0.01 vs. saline-treated group (Tukey’s test)

The results shown in Fig. 5 present glutamate concentration levels, evaluated in the hippocampus during two MRS measurements. The first determination (Fig. 5a) conducted 24 h after a 6-day saline and mephedrone administration cycle showed a statistically significant effect on mephedrone-treated groups (one-way ANOVA: *F* (3, 32) = 6.037, *p* = 0.0022). Moreover, post hoc Tukey’s test showed significant differences in glutamate concentration between saline- and all mephedrone-treated groups (saline vs. mephedrone [5 mg/kg, 10 mg/kg, 20 mg/kg]; *p* < 0.05, *p* < 0.05 and *p* < 0.01, respectively). The second MRS experiment executed 2 weeks after the first one (Fig. 5b) also proved to be statistically significant (one-way ANOVA: *F* (3, 32) = 4.160, *p* = 0.0135). Post hoc Tukey’s test showed statistical differences in glutamate concentrations between saline- and mephedrone-treated (5 mg/kg and 20 mg/kg) animals (*p* < 0.05).

### Chromatographic Determination of Glutamate Concentrations

Table 1 presents chromatographically determined concentrations of glutamate in the hippocampus of rats subjected to 6 days mephedrone administration (5 mg/kg, 10 mg/kg or 20 mg/kg) after 2 weeks of the last mephedrone injection. Statistical analysis did not show significant statistical differences between saline- and mephedrone-treated groups.

**Table 1:** Chromatographic determination of glutamate concentrations in the hippocampus (µM/g tissue)

	Saline	Mephedrone		
		5 mg/kg	10 mg/kg	20 mg/kg
Glutamate concentration (µM/g tissue ± SD)	9.016 ± 0.4	8.182 ± 1.5	8.868 ± 0.61	8.084 ± 0.33

Analysis was performed on the hippocampi of rats subjected to 6 days of mephedrone administration (5–20 mg/kg) and to two in vivo imaging (MRI); *n* = 6. Data represent glutamate concentrations (means ± SD) measured 2 weeks after last mephedrone administration. No significant changes were observed between either of compared groups.

## Discussion

The presented research is the first study undertaken to comprehensively evaluate glutamatergic involvement in rewarding effects of mephedrone using multidisciplinary approach. The behavioural studies were combined with in vivo imaging of glutamate concentrations in the hippocampus using MRS which was subsequently complemented with chromatographic detection of metabolites of interest in animals’ brains. Altogether, the results provide new insight and valid evidence of glutamatergic involvement into mechanisms implicated in rewarding effects of mephedrone.

The rewarding effects of mephedrone, as well as the role of glutamatergic neurotransmission in the expression of these effects, were assessed using the CPP test. CPP is a well-established procedure based on the classical Pavlovian conditioning, which enables to measure the rewarding effects of drugs. Several studies showed that mephedrone elicits rewarding effects in rodents in the CPP model [27–29]. Furthermore, mephedrone has been also shown to produce drug reward in the intracranial self-stimulation (ICSS) test [30–32] and in the self-administration paradigm [20, 21, 33, 34]. In the presented research, it confirmed previously reported rewarding properties of mephedrone in the CPP paradigm and combined it with the evaluation of glutamatergic involvement in the observed effects.

Most drugs of abuse alter glutamatergic transmission in different ways via direct, as well as indirect, interactions with glutamatergic receptors. A strong correlation between glutamatergic neurotransmission and rewarding effects has been proven for many drugs of abuse, e.g. cocaine [35], nicotine [36], alcohol [37] and heroin [38, 39]. Moreover, it has been also reported that pharmacological blockage of glutamatergic transmission attenuates reinforcing effects of drugs [18, for review]. The impact of mephedrone on glutamatergic pathways is still undiscovered. Only one study attempted to evaluate this relationship, showing that mephedrone administration during adolescence induced up-regulation of the GluN2B-containing NMDA receptor in the prefrontal cortex and hippocampus in rats [40]. In our experiment, we evaluated the involvement of glutamatergic neurotransmission in the expression of mephedrone-induced CPP via NMDA receptors using memantine. Memantine is a non-competitive antagonist of NMDA receptors [41] and agonist of dopamine D_2_ receptors [42]. However, it can also act non-selectively and inhibit α-7 nicotinic acetylcholine receptors (nAChRs) [43] and affect 5-HT and DA uptake, as well as sigma-1 receptors and voltage-activated Na channels [44]. It has been shown that memantine does not affect learning [45]; however, it is able to abolish the acquisition [46–48] and expression of cocaine-induced CPP in rodents [46, 48]. Moreover, memantine has been shown to decrease cocaine-induced self-administration [49, 50]. It has also been revealed that memantine abolished the acquisition [51–54] and reinstatement [45, 53, 54] of morphine-induced CPP in mice. Memantine also blocked the acquisition and reinstatement of the MDMA-induced CPP in mice [55].

In the study, a significant behavioural effect of memantine (5 mg/kg) was observed, which blocked the expression of mephedrone-induced CPP. Nevertheless, a decrease in locomotor activity in both saline- and mephedrone-conditioned groups treated with memantine was also observed. This indicates that memantine-induced inhibition of mephedrone-rewarding effects could have been affected by changes in locomotor activity. However, since the mobility of both control and mephedrone-treated groups was affected to the same degree, it may be stated that memantine-induced blockage of mephedrone-induced CPP is likely to be caused by the drug itself, rather than its impact on animals’ mobility. Furthermore, it should be taken into account that memantine can non-selectively affect different central pathways, e.g. via nAChRs or 5-HT and DA neurotransmission [44]. Thus, the limitations of the study that other NMDA-independent mechanisms can also contribute to observed effects of memantine are clear. However, with strong evidence of existing data successfully showing that memantine is able to block rewarding effects of different drugs of abuse and with our MRS results showing an increase in hippocampal glutamate level following mephedrone administration, it can be assumed that glutamatergic neurotransmission is at least partly involved in the expression of mephedrone-induced CPP. Therefore, the presented data can be treated as a promising foundation for further research needed to characterise more precisely the possible involvement of other iGluRs and/or mGluRs in mephedrone-induced expression of drug reward.

Although the reinforcing effects of drugs are associated mainly with an increase in dopaminergic signalling in the drug reward system in the mesocorticolimbic structures, such as NAc, ventral tegmental area (VTA) or PFC, many other pathways and structures also play a significant role in the neurobiology of drug reward. In this research, the assessment of glutamate level was conducted in the hippocampus, a structure of the limbic system which can be affected by drug exposure causing glutamatergic-mediated neuroadaptations [56]. Moreover, it has been shown that drug exposure can lead to the enhancement of the hippocampal function, therefore reinforcing the rewarding effects of drugs of abuse [57]. Additionally, VTA dopaminergic neurons project to the hippocampus mediating emotional and memory responses [56] contributing to the formation of drug-related associations that leads to the development of addiction.

In the study, in vivo MRS showed that 6 days of mephedrone administration increased glutamate hippocampal level in two time points of measurements: 24 h (for the doses of 5 mg/kg, 10 mg/kg and 20 mg/kg) and 2 weeks (for doses of 5 mg/kg and 20 mg/kg) after last mephedrone injection. The fact that increased glutamate concentration in mephedrone-treated animals persisted even for 2 weeks supports the theory that glutamatergic neurotransmission could be considered as a target for developing therapy for mephedrone addiction. So far, only a few studies have tried to evaluate the changes in glutamate brain levels after mephedrone administration. In vivo microdialysis technique revealed increased glutamate release in the NAc and frontal cortex but not in the striatum in adult rats pre-treated with mephedrone in adolescence [19]. Moreover, two complementary ex vivo studies were undertaken to measure mephedrone-related sex-dependent changes in neurotransmitter (i.e. glutamate) levels in the several brain structures, using liquid chromatography mass spectrometry (LC–MS) [20, 21]. These studies revealed that mephedrone exposure decreases glutamate concentration in the thalamus of male rats [20] and increases glutamate concentration in the hypothalamus of female rats [21]; however, no significant changes in the hippocampal glutamate level were observed.

The chromatographic results stay consistent with the above-mentioned ex vivo study that did not show significant differences in the hippocampal glutamate level following mephedrone administration measured post mortem in both male [20] and female [21] rats. However, an additional aim of chromatographic determination of glutamate level in the study was to prove the accuracy of MRS estimations and to demonstrate the validity of MRS as a non-invasive method assessing metabolites’ levels in rodent brains. Although the MRS and chromatographic results are expressed using different units, based on the values of brain tissue density, they can be directly compared. The outcome of the hippocampal glutamate level obtained using both methods is similar; however, the ex vivo results did not show statistically significant difference. Nevertheless, our in vivo MRS measurements were able to detect significant changes between control and mephedrone-treated animals in two time points of measurements. The difference between two methods may appear as the results of physiological changes within the level of glutamate in in vivo and ex vivo tissues and the conditions under which the measurements were performed. Both behavioural and imaging tests are performed on alive individuals, where the brain is washed with cerebral fluid and perfused with blood, which contains and carries neurotransmitters, including glutamate. Therefore, it can be noted that MRS studies quantify the dynamics of cellular metabolism in vivo. However, ex vivo biochemical determinations, which are performed post mortem, are carried out on tissues completely free of body fluids. Furthermore, while preparing tissues for biochemical tests, the brain is rinsed in cold saline, primarily to stop physiological processes as soon as possible as it could negatively affect the biochemical state of the tissue after death, and to get rid of excessive blood. Therefore, biochemical results show the content of glutamate in hippocampal tissue after administration of different doses of mephedrone, while the MRS results depict the change or level of this neurotransmitter during brain work (in this case, the work of the hippocampus).

Furthermore, MR spectroscopy performed in the field strength of 7 T increases the signal-to-noise ratio (SNR) and allows a great precision in the quantification of glutamate, glutamine and gamma-aminobutyric acid [58]. Increased separation and accurate determination of glutamate and glutamine have a high value in diagnosing neurodegenerative and metabolic disorders [59, 60]. MRS is used not only to determine metabolites in central nervous system pathologies but also to observe changes that appear in the addict’s brain [61–64]. Since MRS allows to measure metabolites’ changes in vivo in a non-invasive way, the usage of this technique in behavioural studies has two undeniable advantages. First, it does not require decapitation; therefore, the sacrifice of the animals can be performed in a less stressful way. Secondly, MRS enables to measure time-dependent changes in the same cohort of animals, whereas ex vivo studies would involve multiple animal groups for repeated measurements in different time points of observations. The studies showed that the changes in glutamatergic neurotransmission are long-lasting as we revealed an increase in glutamate levels 2 weeks after the last mephedrone injection.

## Conclusions

Altogether, the comparison between behavioural and in vivo MRI studies gave new, complex insight into mechanisms underlying the expression of mephedrone-induced CPP, indicating that glutamate neurotransmission is involved in rewarding effects of mephedrone. Moreover, mephedrone-induced changes in glutamate levels in the hippocampus are long-lasting, which should be of particular importance while developing new strategies in the treatment of mephedrone addiction. In addition, a comparison of the results from MRI and chromatographic studies proved the validity and utility of the MRS method in behavioural and neuropsychopharmacological research. The presented research can successfully serve as a foundation for further studies undertaken to explore in detail glutamatergic involvement (via other subtypes of GluRs) in mephedrone-induced expression of drug reward in animal models.

## Supplementary Information

Below is the link to the electronic supplementary material.

Supplementary file1 (DOCX 14 KB)

## Data Availability

Raw data are available from the corresponding author on reasonable request.
