# Supplementary material for: Insight into Glutamatergic Involvement in Rewarding Effects of Mephedrone in Rats: In Vivo and Ex Vivo Study
Source: Mol Neurobiol. 2021 May 21;58(9):4413–24. doi: 10.1007/s12035-021-02404-y (PMC8487417; doi:10.1007/s12035-021-02404-y)
Supplement: Supplementary file 1 — Supplementary file1 (DOCX 14 KB) [file 12035_2021_2404_MOESM1_ESM.docx]

**Detailed description of Chronic Mild Unpredictable Stress (CMUS) paradigm**

***Applied stressors***

1) lack of litter for 24 h,

2) damp sawdust for 24 h,

3) swimming in cold water (13° for 5 min),

4) tilted cage at 45° for 4 h,

5) lights on overnight,

6) food deprivation for 24 h,

7) water deprivation for 12 h,

8) cage shaking (4 times for 2 min in 5 min interval),

9) unpleasant sound (4 times for 5 min in 10 min interval).

***Schedule***

| ***Day 1*** | Water deprivation | ***Day 12*** | Swimming in cold water |
| --- | --- | --- | --- |
| ***Day 2*** | Lack of litter | ***Day 13*** | Food deprivation |
| ***Day 3*** | Tilted cage | ***Day 14 (Habituation)*** | Damp sawdust |
| ***Day 4*** | Unpleasant sound | ***Day 15 (Pre-test)*** | Cage shaking |
| ***Day 5*** | Swimming in cold water | ***Day 16 (Conditioning)*** | Unpleasant sound |
| ***Day 6*** | Lights overnight | ***Day 17 (Conditioning)*** | Lack of litter |
| ***Day 7*** | Food deprivation | ***Day 18 (Conditioning)*** | Lights overnight |
| ***Day 8*** | Cage shaking | ***Day 19 (Conditioning)*** | Water deprivation |
| ***Day 9*** | Damp sawdust | ***Day 20 (Conditioning)*** | Unpleasant sound |
| ***Day 10*** | Lack of litter | ***Day 21 (Conditioning)*** | Cage shaking |
| ***Day 11*** | Tilted cage | ***Day 22 (Test)*** | No stressor |

***Detailed schedule of the applied stressors throughout the CPP test***

**Day 14**: 15 min habituation → 1h → Damp sawdust 24 h

**Day 15**: Come back to the regular cages → 1h → Pre-test (15 min) → 1 h → Cage shaking

**Day 16:** Conditioning: Morning session → 4 h **→** Afternoon session → 1 h **→** Unpleasant sound

**Day 17**: Conditioning: Morning session → 4 h **→** Afternoon session → 1 h → Lack of litter

**Day 18:** Conditioning: Morning session → 4 h **→**Afternoon session → 1 h → Come back to the regular cages → 8.00 p.m. Lights overnight

**Day 19:** Conditioning: Morning session → 4 h **→** Afternoon session → 8.00 p.m. Water deprivation (water was given back to rats 30 min before the next day’s morning session)

**Day 20:** Conditioning: Morning session → 4 h **→** Afternoon session → 1h → Unpleasant sound

**Day 21:** Conditioning: Morning session → 4 h **→** Afternoon session → 1h → Cage shaking

**Day 22:** Test (no stressor)
